# Superparasitism Drives Heritable Symbiont Epidemiology and Host Sex Ratio in a Wasp

**DOI:** 10.1371/journal.ppat.1005629

**Published:** 2016-06-20

**Authors:** Steven R. Parratt, Crystal L. Frost, Martijn A. Schenkel, Annabel Rice, Gregory D. D. Hurst, Kayla C. King

**Affiliations:** Institute of Integrative Biology, University of Liverpool, Liverpool, United Kingdom; University of Cambridge, UNITED KINGDOM

## Abstract

Heritable microbial symbionts have profound impacts upon the biology of their arthropod hosts. Whilst our current understanding of the dynamics of these symbionts is typically cast within a framework of vertical transmission only, horizontal transmission has been observed in a number of cases. For instance, several symbionts can transmit horizontally when their parasitoid hosts share oviposition patches with uninfected conspecifics, a phenomenon called superparasitism. Despite this, horizontal transmission, and the host contact structures that facilitates it, have not been considered in heritable symbiont epidemiology. Here, we tested for the importance of host contact, and resulting horizontal transmission, for the epidemiology of a male-killing heritable symbiont (*Arsenophonus nasoniae*) in parasitoid wasp hosts. We observed that host contact through superparasitism is necessary for this symbiont’s spread in populations of its primary host *Nasonia vitripennis*, such that when superparasitism rates are high, *A*. *nasoniae* almost reaches fixation, causes highly female biased population sex ratios and consequently causes local host extinction. We further tested if natural interspecific variation in superparasitism behaviours predicted symbiont dynamics among parasitoid species. We found that *A*. *nasoniae* was maintained in laboratory populations of a closely related set of *Nasonia* species, but declined in other, more distantly related pteromalid hosts. The natural proclivity of a species to superparasitise was the primary factor determining symbiont persistence. Our results thus indicate that host contact behaviour is a key factor for heritable microbe dynamics when horizontal transmission is possible, and that ‘reproductive parasite’ phenotypes, such as male-killing, may be of secondary importance in the dynamics of such symbiont infections.

## Author Summary

Most insects house heritable symbionts and these represent an important component of their biology, both as partners conveying beneficial traits such as defence against natural enemies, or as antagonists manipulating their hosts’ reproduction. Work on these bacteria mostly assumes that such phenotypes have evolved primarily to facilitate the symbiont’s vertical transmission from parent to offspring. However, several such bacteria also move horizontally between unrelated individuals. Here, we show that a male-killing symbiont actually depends upon horizontal transmission for its spread and maintenance. We observed *Arsenophonus nasoniae* was only maintained in parasitoid wasp populations when the route enabling horizontal transmission, superparasitism of fly pupae, was allowed. When superparasitism was common enough to cause epidemic spread of *A*. *nasoniae*, host population extinction occurred due to lack of males. Our study indicates that superparasitism behaviour is likely to be the key element determining which wasp species maintain this symbiont in nature. This provides new insights into the factors determining heritable symbiont frequency within and amongst species, and highlights the extreme effects such symbionts can have on their host populations. The data also indicate that male-killing may evolve and be maintained as an additional, rather than primary, driver of heritable symbiont fitness.

## Introduction

Heritable symbionts are common in natural populations of arthropods [1], where they affect the biology of their host individual in diverse ways. They can be obligatory, providing physiologically crucial functions to their host, such as amino acid or vitamin anabolism [2], or alternatively provide ecologically contingent benefits, such as conferring the ability to resist natural enemy attack [3,4]. Finally, they can be parasitic, spreading through their host population via the distortion of host reproductive biology towards the production and survival of infected females [1]. These impacts on the individual host can have consequences for ecology and evolution at the host population level when the symbionts spread efficiently [5–9]. Thus, it is important to understand the factors that contribute to symbiont epidemiology.

Past work on heritable symbiont epidemiology has emphasized vertical transmission (VT) through maternal inheritance as the dominant means by which new infections are established [10–16]. Within this framework, aspects of host ecology, such as contact structure, are commonly ignored as they are not thought to influence microbe transmission. However, a number of heritable microbes that infect insects readily combine VT with horizontal transmission (HT), creating symbionts with a mixed-mode of transmission [17]. Unlike VT, HT rates are dependent on the degree of contact between hosts and thus may represent a means through which host ecology and behaviour can drive symbiont spread. However, HT and host contacts have not been empirically explored in heritable symbiont epidemiology.

Heritable symbionts can horizontally transmit via several mechanisms, including passing through the phloem of a host’s food plant [18], being vectored via parasitoid wasp ovipositors [19,20], or by being transmitted when mating [21]. Notably, several symbionts that infect parasitoid wasps achieve HT when infected and uninfected host females share an oviposition target–a phenomenon termed superparasitism when occurring between females of the same species, and multiparasitism when a host is shared by two or more parasitoid species [22–26]. For heritable microbes such as these, the contact structure of the host is likely to be an important determinant of symbiont dynamics, as each contact represents a transmission opportunity. Specifically for parasitoid species, superparasitism rates will impact on the probability of acquiring a symbiont, thus linking host reproductive behaviour and symbiont epidemiology. Remarkably, this host behavioural trait has also been shown to be manipulated by a virus to promote its HT [25,26].

Here, we use an experimental epidemiology approach to directly evaluate the impact of host contact structure on the dynamics of a reproductive parasite with mixed-mode transmission. We manipulate the host contact structure (opportunity for superparasitism) in populations of parasitoid chalcid wasps and subsequently track the dynamics of the male-killing bacteria *Arsenophonus nasoniae*. *A*. *nasoniae* was originally described in the wasp *Nasonia vitripennis*, where it kills *c*.80% of the male offspring of infected females [23,27–29]. *A*. *nasoniae* is unusual among male-killers as it is not directly transmitted within the host’s egg. Rather, this bacterium achieves VT when a female wasp oviposits into a fly pupa and the bacterium (which is extracellular) is inoculated into the fly host with the wasp’s venom. *A*. *nasoniae* then infects the wasp larvae that hatch as they feed on the fly cadaver [30]. If an uninfected and infected female *N*. *vitripennis* superparasitise a fly pupa, the progeny of both females become infected, creating horizontal transmission [23,24]. Despite this capacity for HT, the dynamics of *A*. *nasoniae* and other male-killers have only been described with VT-only models, wherein male death increases the fitness of their infected sisters; a mechanism termed ‘fitness compensation’ [10,11]. Empirical evidence for such benefits in this system is weak and/or lacking [31], indicating other factors may be key for *A*. *nasoniae* spread.

We further explore whether host contact behaviours, and thus opportunities for HT, can influence symbiont dynamics in multiple, related host species. *N*. *vitripennis* shares its filth-fly niche with a number of other parasitoids that can acquire the symbiont through interspecific multiparasitism in the laboratory [24]. However, surveys of natural parasitoid communities have found little or no *A*. *nasoniae* infection in many of these species [24,32]. We hypothesise that the ability of *A*. *nasoniae* to spread and be maintained in new parasitoid host species is dependent upon HT through superparasitism. In these multi-species experiments, we track symbiont dynamics, VT efficiency and cost of infection in a number parasitoid species that vary in their superparasitism propensity. From this we determine the potential importance of superparasitism behaviour in driving variable patterns *A*. *nasoniae* dynamics between host species.

## Results

### 1. Opportunity for superparasitism affects *A*. *nasoniae* dynamics in *N*. *vitripennis*

We first compared the dynamics of *A*. *nasoniae* in populations of *N*. *vitripennis* when superparasitism was permitted compared to populations where solitary parasitism was enforced. Following this, we examined quantitatively the impact of different opportunities for superparasitism (i.e. varying degrees of host contact) on *A*. *nasoniae* dynamics. Our hypothesis was that host contact through superparasitism would drive *A*. *nasoniae* into populations through HT, and that higher rates of superparasitism would be associated with higher prevalence of the symbiont. Within this, we also investigated whether pupal host resource level (number of fly pupae offered per wasp) influenced *A*. *nasoniae* dynamics.

#### Superparasitism is required for symbiont maintenance

We first tested if superparasitism was required for *A*. *nasoniae* maintenance. We established replicated laboratory populations of *N*. *vitripennis* wasps in which 50% of individuals initially carried the male-killing symbiont, and which differed in the presence/absence of superparasitism opportunity. To this end, populations of 80 female wasps were established and split into four treatments in a 2×2 factorial design: superparasitism always/never available (four females per patch vs one); high or low host resources (four or one fly pupae per wasp, respectively). Each of these four conditions was six-fold replicated and maintained over eight generations with only local mating permitted (mirroring the natural biology of the system). The number of female wasps founding each generation was held at 80 across all treatments to control against drift effects. The prevalence of *A*. *nasoniae* and host population sex ratio was scored each generation.

**Fig 1:**
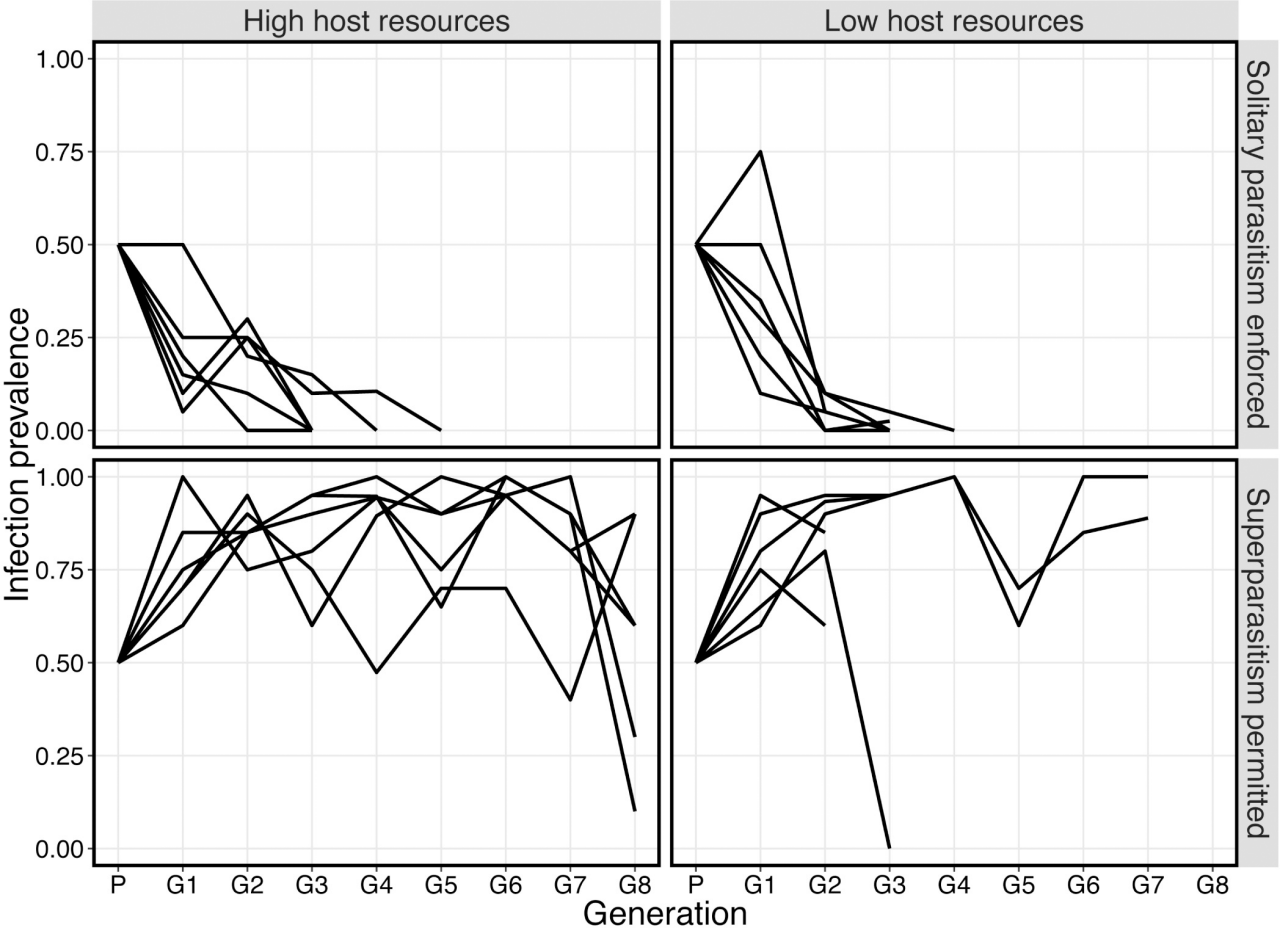
Mean infection prevalence of *A*. *nasoniae* in replicate populations of *N*. *vitripennis* over eight generations. All parental populations were started with 80 females, 50% of which were infected with *A*. *nasoniae*. Infection was lost by generation five in all populations in which solitary parasitism was enforced, but increased and persisted when superparasitism was allowed.

When solitary parasitism was enforced (no host contacts) *A*. *nasoniae* was lost from the population in less than five generations for both high and low pupal resource treatments (all replicates, *n* = 12). In contrast, *A*. *nasoniae* spread rapidly and was maintained at high prevalence in 11 of 12 populations in which superparasitism was permitted (Fig 1). To retain a balanced design in our analyses, infection prevalence was analysed at the second generation, in which all replicate populations were still viable and infection had not been lost in any treatments. At this time point, infection prevalence was significantly higher when superparasitism was permitted (comparison of binomial GLMMs, χ^2^ = 48.527, df = 1, *P*<0.001), and was marginally, negatively correlated with low host-resource levels (comparison of binomial GLMMs, χ^2^ = 4.245, df = 1, *P* = 0.039).

#### High symbiont prevalence causes fluctuations in population sex-ratio and local extinction

**Fig 2:**
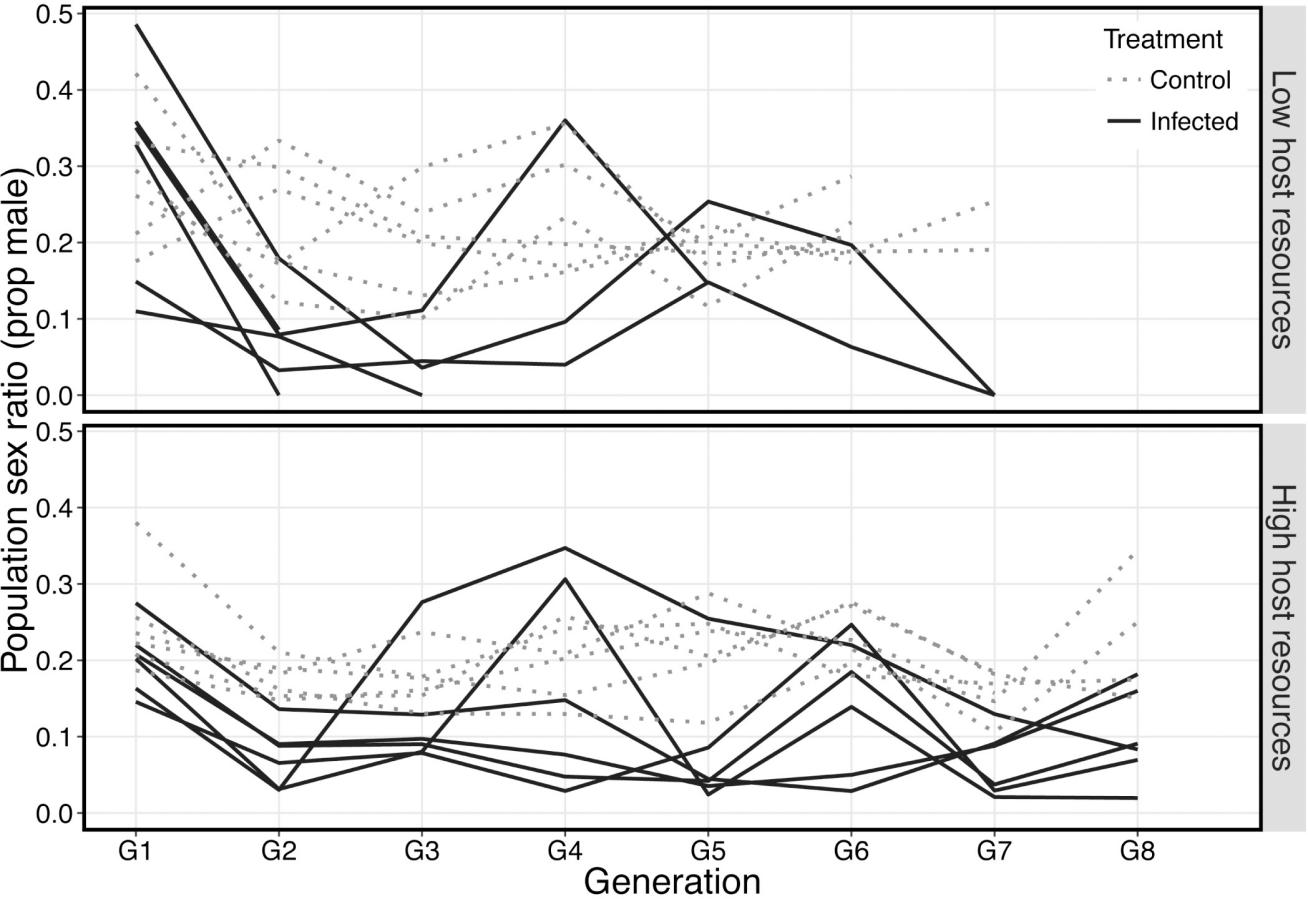
Sex ratio (proportion male) of populations of *N. vitripennis* in which superparasitism was permitted and host resources availability was high (top panel) or low (bottom panel). Black lines are individual replicate populations that were infected with *A*. *nasoniae*, grey lines are replicate control populations. *A*. *nasoniae* infection and spread caused significant deviation in sex ratio compared to uninfected controls (See Table 1). Furthermore, sex ratio varied considerable among infected populations in the same treatment group in many generations. Each treatment was run in two separate blocks of 3 replicates of both control and infected populations. Control populations were no longer maintained when all of their contemporary infected populations either lost the infection or went extinct.

**Fig 3:**
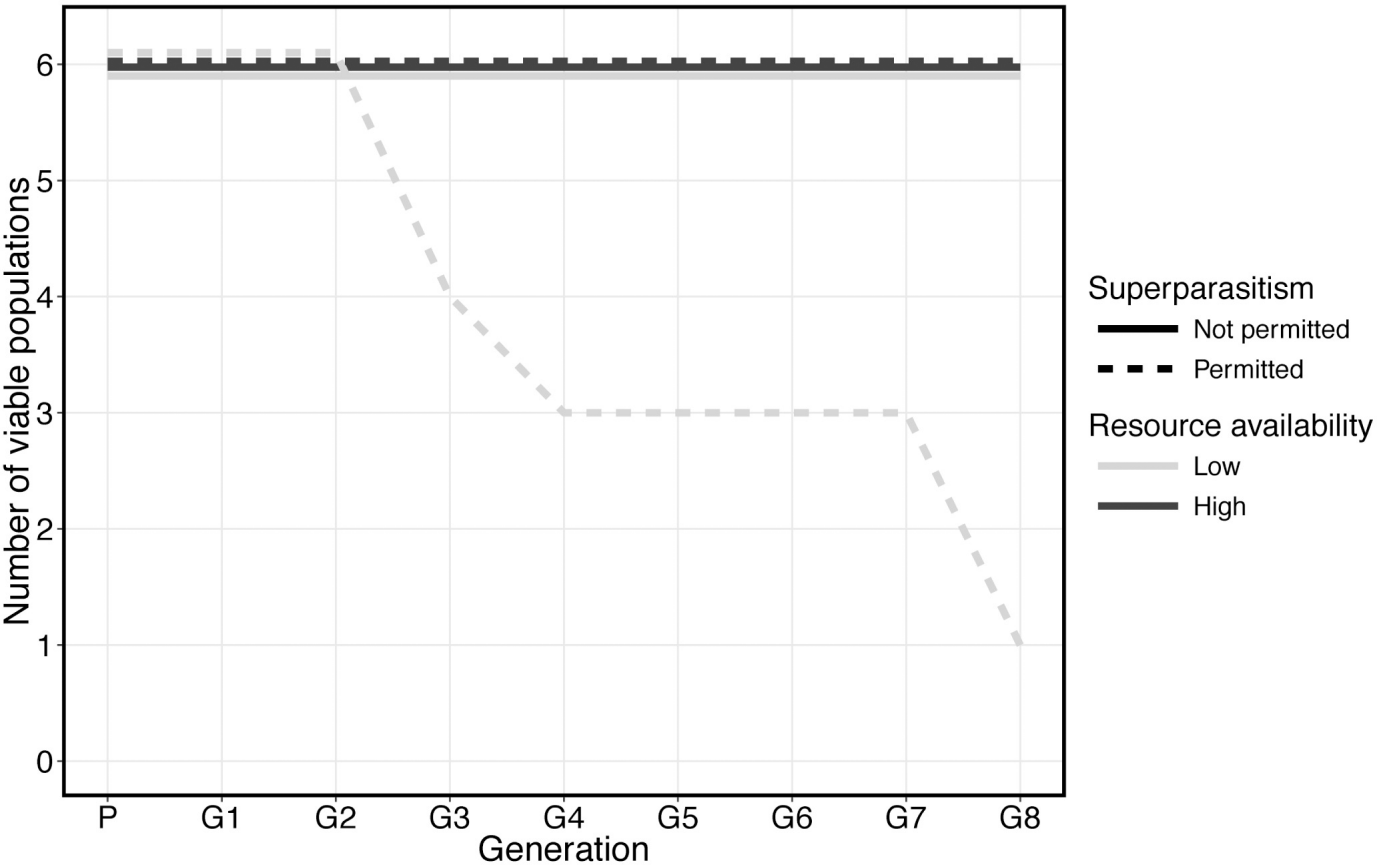
Viability of replicate populations of *N*. *vitripennis* over the course of the experiment. Populations in which *A*. *nasoniae* was purged are considered extant. Uninfected control populations not included.

In treatments where superparasitism drove infection to high prevalence we also observed significant decreases in population sex ratio compared to uninfected controls (Fig 2 and Table 1). This effect varied in severity between generations and was asynchronous among replicate populations. Furthermore, five replicate populations in which infection increased and fly resources were low went extinct, most likely due to these sex ratio fluctuations. At generation eight, population extinction was significantly associated with the opportunity for wasps to superparasitise (Fisher’s exact test, *P* = 0.013), and low host resource (Fisher’s exact test *P* = 0.013)(Fig 3). Only a single uninfected control population under the same fly and parasitoid densities went extinct. This strongly indicates that symbiont induced sex-ratio bias is the major factor in eliciting host population crashes (control vs infected populations: Fisher’s exact test *P* = 0.015).

**Table 1:** Differences in mean sex ratio (proportion male) between symbiont infected populations of *N*. *vitripennis* and corresponding uninfected controls (negative values represent more female biased sex ratios).

Generation	Low host resource, superparasitism permitted			High host resource, superparasitism permitted		
	Effect of infection on sex ratio relative to control.	Significance level	Notes	Effect of infection on sex ratio relative to control.	Significance level	Notes
G1	0.04	NS		-0.26	NS	
G2	-1.4	***		-0.89	***	
G3	-1.89	***	Two populations extinct	-0.43	NS	
G4	-0.63	NS	One population extinct, one purged.	-0.54	NS	
G5	-0.02	NS		-1.18	***	
G6	-	-	Very small, all-female broods.	-0.52	***	
G7	-	-	All extinct	-1.1	***	
G8	-	-	All extinct	-1.31	***	

Data shown for treatments where superparasitism was permitted. The difference in sex ratio fluctuates from highly significant to non-significant. This is a product of demographic instability caused by male-killing and the production of all male broods by virgin females. All statistics are comparisons of GLMERs with/without fixed effect of treatment.

^a^*** = *P*<0.001, NS: P>0.05.

#### Symbiont prevalence positively correlates with population-level superparasitism frequency

We then examined the effect of within-population variation in superparasitism opportunity on *A*. *nasoniae* dynamics. To this end populations of *N*. *vitripennis* with varying degrees of superparasitism opportunity (0, 10, 20, 30, 50, 100% of wasps parasitizing in groups) were established and infection prevalence scored after four generations of propagation. All populations started at 50% *A*. *nasoniae* prevalence and high resource levels (1 fly pupae per female in a patch) with 40 female wasps per population.

**Fig 4:**
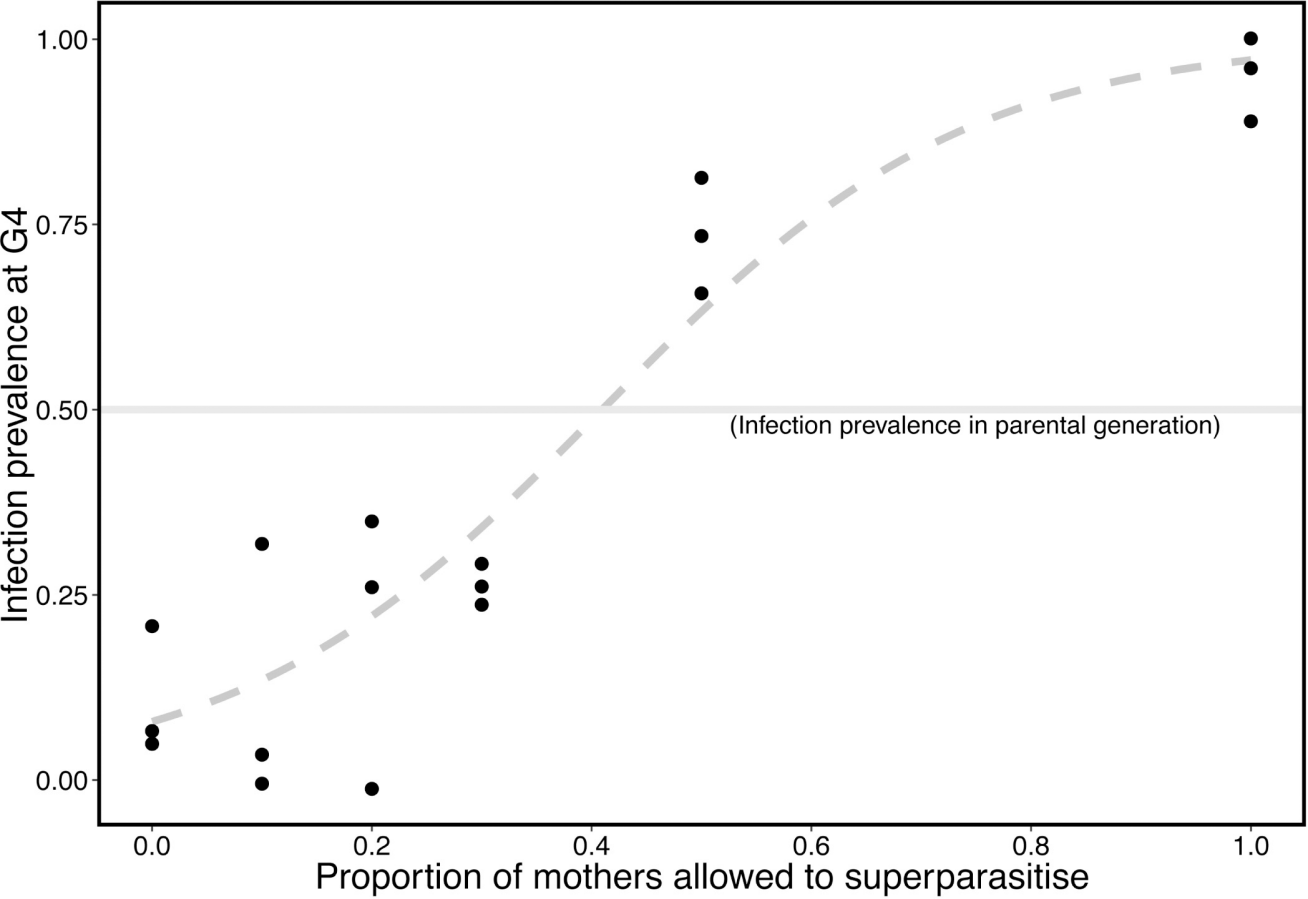
Prevalence of *A*. *nasoniae* in populations of *N*. *vitripennis* kept under varying opportunities to superparasitise for four generations. All populations were started with 50% prevalence of *A*. *nasoniae* (dotted lines). The trend line is fitted from predictions of a binomially distributed GLM.

Infection prevalence after four generations was observed to be significantly, positively associated with superparasitism opportunity (comparison of binomial GLMMs, χ^2^ = 30.154, df = 1, *P*<0.001, Fig 4). In all cases the final infection prevalence significantly deviated from the starting prevalence of 50%. Prevalence increased where 50% or more wasps had the opportunity to superparasitise, and reduced where superparasitism was possible for 30% or fewer wasps (exact binomial test, *P* <0.001 in all cases, Fig 4).

### 2. Persistence of *A*. *nasoniae* is lower in non-*Nasonia* hosts and is associated with reduced tendency to superparasitise

*N*. *vitripennis* shares its filth fly niche with several other parasitoids, including *N*. *longicornis*, *N*. *giraulti*, *Trichomalopsis sarcophagae* and *Muscidifurax raptorellus* [33]. Previous work has shown that *N*. *longicornis*, *N*. *giraulti* and *M*. *raptorellus* can acquire *A*. *nasoniae* when multiparasitising with infected *N*. *vitripennis* under controlled conditions [24], though this mode of interspecific transfer has not been previously tested with *T*. *sarcophagae*. In nature, all *Nasonia* species used here maintain the symbiont, but it has not been found in surveys of *M*. *raptorellus* or *T*. *sarcophagae* [24,32].

We examined the dynamics of *A*. *nasoniae* in these parasitoid wasp species to investigate whether close relatedness of a wasp species to *N*. *vitripennis* predicted *A*. *nasoniae* maintenance and to determine the biological basis of the pattern. We established replicated populations for each of the five wasp species at 100% initial *A*. *nasoniae* prevalence. Superparasitism was permitted in all populations.

#### Phylogeny predicts symbiont persistence

**Fig 5:**
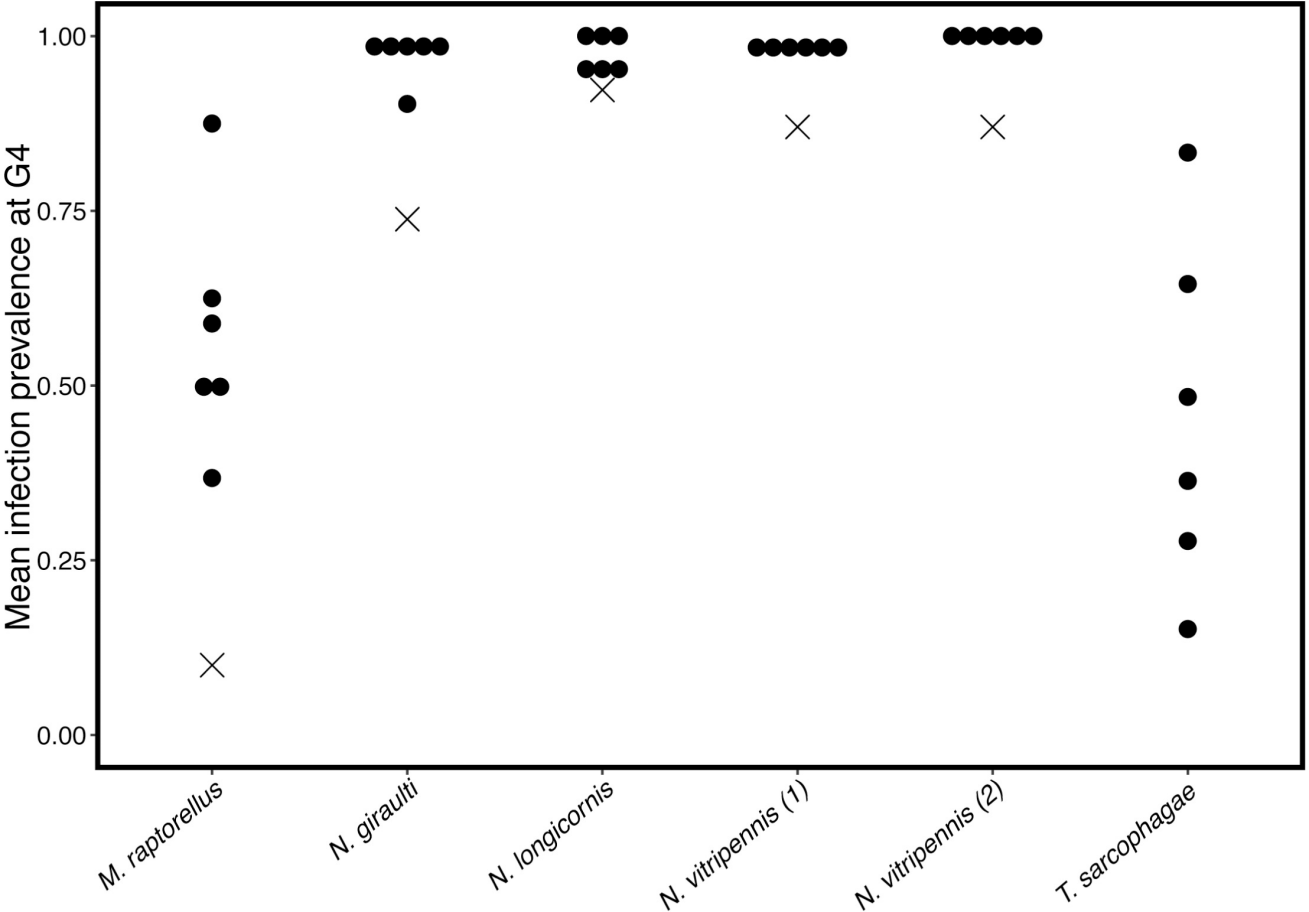
Prevalence of *A*. *nasoniae* in populations of five parasitoid wasp species given opportunity to superparasitse for four generations. All *Nasonia* species showed significantly higher infection prevalence than non-*Nasonia* in both rounds (all contrasts *P*<0.001). All other comparisons showed no significant difference in infection. ‘X’s indicate predicted prevalence of the symbiont if only vertical transmission occurred. Observed values are significantly higher than the predictions in all cases (Exact binomial tests *P* = 0.02) for all species for which the epidemiological model could be parameterized.

We observed that *A*. *nasoniae* was maintained at high prevalence in the three species from the *Nasonia* complex but declined in *M*. *raptorellus* and *T*. *sarcophagae* (species effect on prevalence GLMMs, χ^2^ = 97.294, df = 5, *P* <0.001, all pairwise comparison between *Nasonia* spp and others *P*<0.001) (Fig 5).

#### Vertical transmission efficiency and cost of infection do not explain observed A. nasoniae dynamics in multiple species

**Fig 6:**
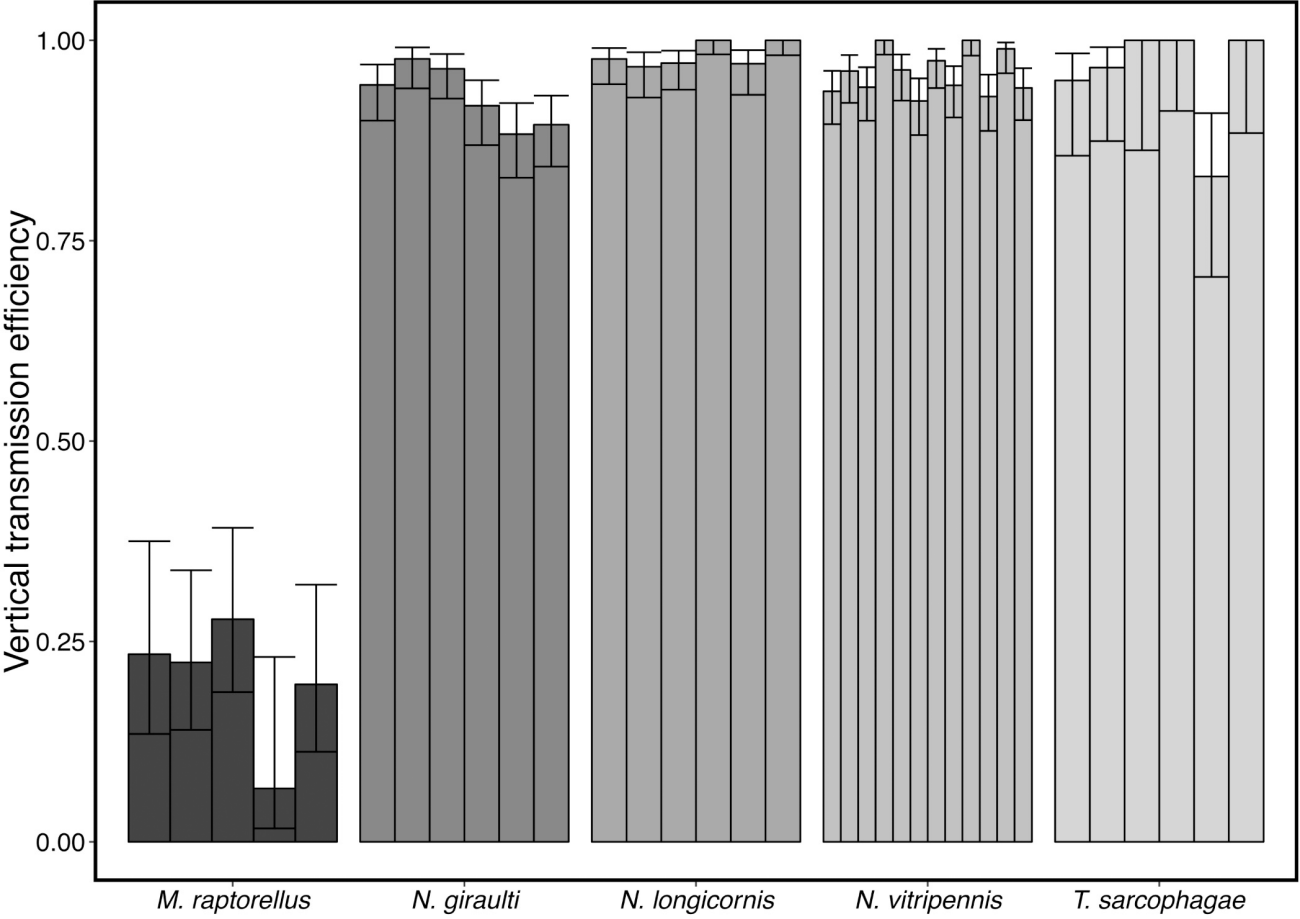
Vertical transmission efficiency of *A*. *nasoniae* as measured in single-female oviposition assays from replicate populations of five parasitoid wasp species. VT efficiency was significantly reduced in *M*. *raptorellus* compared with all other species (all pairwise contrasts *P*<0.001). Bars = 95%CI calculated with logit link for proportional data.

Variation in VT efficiency and symbiont-induced effects on daughter production were measured in each of the wasp species. No significant differences in VT rate were found between the three *Nasonia* species and *T*. *sarcophagae*. However, *A*. *nasoniae* showed significantly lower VT rates in *M*. *raptorellus* compared to the other four species and represents an important source of the failure of the symbiont to persist in this species (Tukey contrasts from GLMER all *P*<0.001) (Fig 6). In addition, we found no association between *A*. *nasoniae* VT efficiency and the mean number of daughters produced by these species.

**Fig 7:**
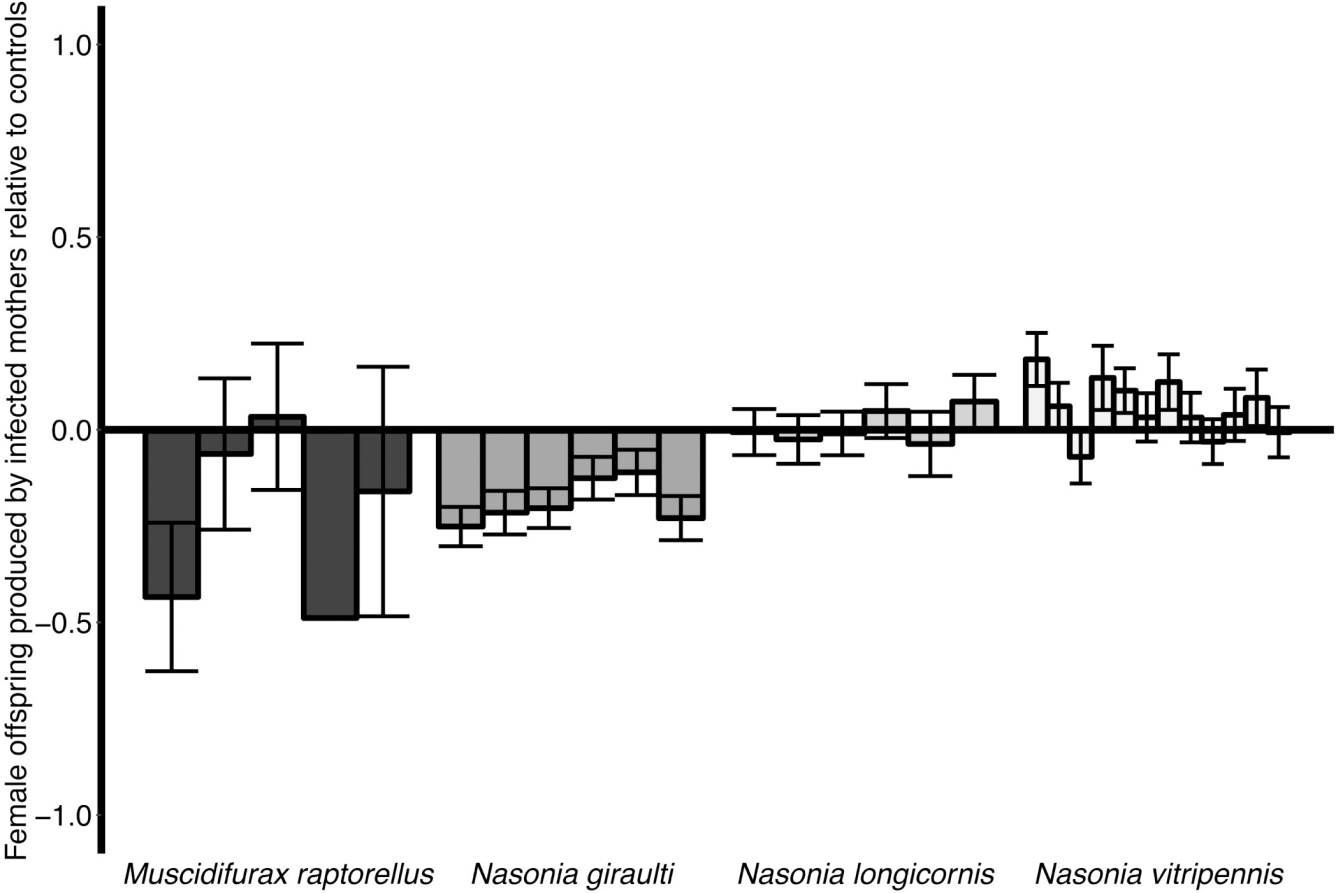
Impact of *A*. *nasoniae* carriage on the number of daughters produced in four of the five parasitoid species. Negative values represent replicates where infected females produce fewer adult daughters compared to uninfected controls. Daughter production was only significantly lower in one species, *N*. *giraulti* (GLMER, χ^2^ = 6.6437, df = 1, *P* = 0.009). The fifth species, *T*. *sarcophagae* is not included due to absence of uninfected control populations for comparison. Bars = ±1 SE.

Infection cost was measured as the number of daughters produced by infected females relative to uninfected females of the same species. *A*. *nasoniae* infection only affected daughter production in *N*. *giraulti*, where it significantly reduced the number of daughters emerging from a pupa compared to uninfected control individuals (GL*MER*, χ^2^ = 6.64, *df = 1*, *P* = 0.009, Fig 7). However, this species was competent to maintain infection, indicating that HT was sufficient to overcome costs of infection in our populations. Daughter production was not measured for one species, *T*. *sarcophagae*, due to a population crash in the uninfected control populations.

We used our observations of VT efficiency and infection cost to parameterize a simple epidemiological model of *A*. *nasoniae* dynamics, in which we assume no HT occurs (crosses, Fig 5). The observed infection prevalence after four generations was significantly higher in each host species than that predicted by the VT only model (exact binomial tests all *P*≤0.002). This observation is consistent with the need for horizontal transmission, facilitated through superparasitism, to explain our observed prevalence of *A*. *nasoniae* in each of these species, even when accounting for poor vertical transmission or cost.

#### Interspecific variation in superparasitism behaviour is associated with host competence for maintaining *A*. *nasoniae*

Finally, we linked the likelihood of the different species to superparasitise with the competence of those species to maintain *A*. *nasoniae*. We performed behavioural assays of females to measure superparasitism avoidance behaviour in the rearing conditions used within populations for each of the five species, *N*. *vitripennis*, *N*. *giraulti*, *N*. *longicornis*, *T*. *sarcophagae* and *M*. *raptorellus*.

**Fig 8:**
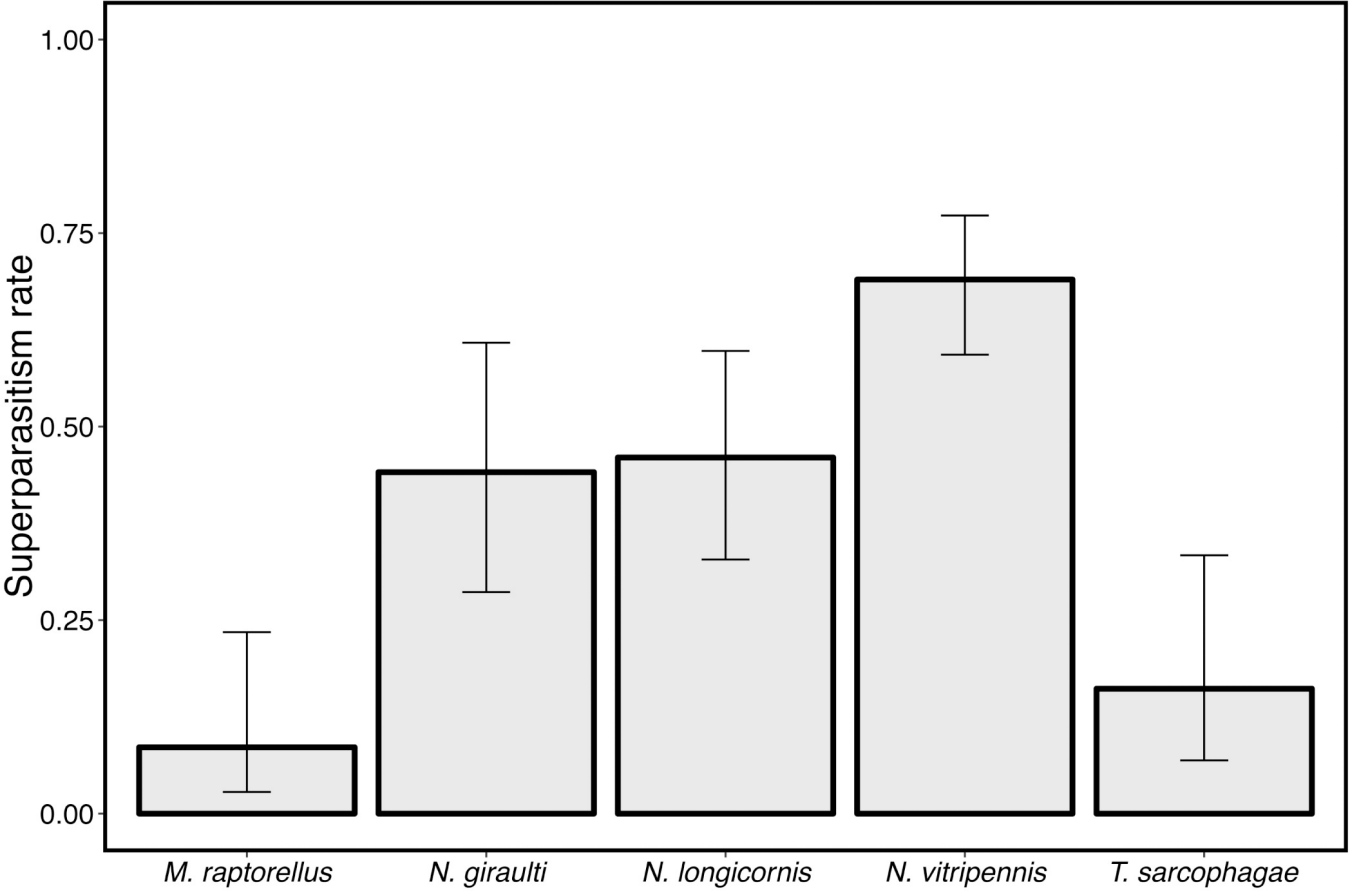
Superparasitism rates of five parasitoid species. Wasps in the *Nasonia* complex had significantly higher rates of superparasitism than either *M*. *raptorellus* or *T*. *sarcophagae* (Pairwise contrasts of all *Nasonia* spp vs others *P<*0.001, see S1 Table). Bars are 95% CI calculated with logit link for binomial data.

These assays revealed that wasp species significantly differed in the likelihood of two individuals using the same pupa (comparisons of binomial GLMs χ^2^ = 101.08, df = 4, *P*<0.001). Wasps in the *Nasonia* complex had significantly higher rates of superparasitism than either *M*. *raptorellus* or *T*. *sarcophagae*, which both showed significantly lower levels of superparasitism (Fig 8, pairwise contrasts in full S1 Table). Reduced superparasitism by the host corresponds to lower opportunity for *A*. *nasoniae* to transmit horizontally in these two species. This suggests that superparasitism avoidance contributes to poor *A*. *nasoniae* maintenance of these host species.

## Discussion

The ability of heritable symbionts to horizontally transmit is increasingly being appreciated [18–22,24,26,34,35], but its importance in symbiont epidemiology within host populations has not been empirically investigated. Here, we show that host-host contact during superparasitism, a behaviour that permits HT in our system, is necessary for the spread of a heritable male-killer. Further, the extent of symbiont spread relates closely to the frequency of host contacts in the population. We also demonstrate that variation in symbiont maintenance between host species may be partly explained by the differing superparasitism behaviours exhibited between wasp species. These results have implications for our understanding of heritable symbiont epidemiology, the evolution of reproductive manipulation and the consequences of superparasitism in parasitoids.

In our experiments, *A*. *nasoniae* was lost from wasp populations unless HT was permitted through superparasitism. This suggests that the dynamics of symbionts with mixed-mode transmission are strongly reliant upon host contact rates. From this observation, we predict that variation in *A*. *nasoniae* prevalence across host populations and species will be driven by both variation in contact rates/behaviours, and by the reproductive rate of infected females as presumed under VT-only models. For example, for species such as *N*. *vitripennis*, wasp density (and therefore superparasitism intensity) vary greatly over population ranges [36,37], with up to 40% of fly pupae being parasitized by more than one female wasp [38]. Our results suggest that this variation will impact on the frequency of *A*. *nasoniae* infection. These findings echoes theoretical and field studies of an insect virus that exhibits a similar mixed-mode of transmission [39,40], in which spatially heterogeneous host and parasitoid densities have been linked with variation in symbiont prevalence.

Because HT breaks down the link between host relatedness and symbiont transmission, our findings present an interesting exception to the current view on the spread of male-killing symbionts. Under VT-only models, male-killing is thought to be most common when competition is between siblings (i.e. no superparasitism), as the death of males directly benefits the fitness of their infected sisters and thus promotes symbiont VT (fitness compensation) [10,11,41]. Our observation that *A*. *nasoniae* is lost from host populations when superparasitism is prevented indicates that reproductive parasitism phenotypes alone are not sufficiently strong to maintain infection in this system. Indeed, *A*. *nasoniae* was maintained in *N*. *giraulti*, which readily superparasitizes, despite an observed 10–20% cost of symbiont carriage, measured in terms of daughter production. Furthermore, our VT-only model predicts higher symbiont prevalence after 4 generations than we see in our populations of *N*. *vitripennis* kept under solitary parasitism conditions. This result may indicate that *A*. *nasoniae* is exerting an additional cost on its host that we do not detect and allows us to exclude any advantage of male-killing as sufficient for maintenance of *A*. *nasoniae* in these species.

The observation that there is only a weak benefit of male-killing to symbiont transmission is echoed in other studies of male-killing [15,31,42]. In some instances such ‘weak’ male-killing microbes have also additional phenotypes such as protecting their host against natural enemy attack [15,43]. Furthermore, where male killing efficiency is compromised by host evolved suppression, additional reproductive manipulation phenotypes have been revealed [44]. Our results, in combination with these previous studies, indicate that male-killing may evolve and be maintained as an additional, rather than primary, driver of heritable symbiont fitness in a number of cases. Unlike these other systems where additional phenotypes also promote VT, our findings show that for *A*. *nasoniae* any benefit of male-killing is supplementing obligate HT. Indeed, *A*. *nasoniae* is unusual among male killers in that it may be grown in cell-free culture and requires HT in order to persist as we have revealed. As such, male-killing in *A*. *nasoniae* may have arisen through an entirely different evolutionary trajectory to that in more ‘traditional’ heritable symbionts where male-killers show greater linkage with their host line [45,46]. Nevertheless, we would note that the use of HT in addition to sex ratio distortion is not unique to our system, but is additionally shown by parthenogenesis inducing *Wolbachia* in trichogrammatid wasps [22], although the dynamical importance of HT has not been determined in this system.

Our experiments and measurements beg the question as to why male-killing has been maintained or evolved at all in this system. We suggest that although fitness compensation whilst is not sufficient to enable spread of the symbiont on its own, it provides enough of a marginal benefit to be advantageous over non-male-killing mutants. Indeed, contrary to the current paradigm that male-killing is most favoured when competition is primarily between siblings[12], the impact of male-killing on *A*. *nasoniae* transmission may actually be stronger under HT than under VT-only. This arises because resource competition in a superparasitised pupae will likely be more intense due to crowding [47], and the infection transfers to both sibships within the host pupa. Balas *et al* [31] proposed a verbal model of a similar mechanism called the ‘incremental gains hypothesis’ to explain the marginal benefits of male-killing observed in wild caught *A*. *nasoniae*-infected wasps. The data presented here empirically support this otherwise neglected model. Interestingly, this predicts that solitary parasitoids should be less likely to harbour male-killing, horizontally transmitted symbionts because there are no brood-mates to horizontally transmit into. Indeed, to our knowledge, the male-killing phenotype has not been described in any symbiont infection of a solitary parasitoid.

We observed that *A*. *nasoniae* maintenance varied between parasitoid species, an outcome that correlated positively with variation in superparasitism rates. This result strongly supports the hypothesis that superparasitism propensity may contribute towards the persistence of *A*. *nasoniae* after host shift events. Host-symbiont interactions in novel hosts may be disrupted compared to that in ancestral hosts in a variety of ways, e.g. by increased infection costs or low VT efficiency [48,49]. These disruptions may prevent spread. In contrast, we observed maintenance of the infection in *N*. *giraulti*, a host which readily superparasitised, despite a considerable cost to infection. Thus, high rates of superparasitism are able to compensate for a symbiont misfit and permit symbiont maintenance.

In our experiments, individuals were maintained under conditions in which superparasitism would be necessary if every female were to oviposit. Thus they represent an ecological extreme of host parasitoid density and fly pupal scarcity that leaves only wasp behavioural variation as the determinant of superparasitism rate. In nature, spatiotemporal heterogeneity in wasp and fly densities are likely to be the major determinants of superparasitism rate and, so too, *A*. *nasoniae* epidemiology in these species. This effect has been explored both in theoretically and field studies of LbFV where viral prevalence is low or absent in less dense populations at the host-species range [39,40]. We thus argue that once extrinsically determined host contact networks are accounted for, the intrinsic effect of host propensity to superparasitise may play a role in the host range for this symbiont in nature. Potentially, certain arthropod groups may act as hotspot reservoirs of symbiont infection by virtue of their oviposition behaviour.

Our results also add a new facet to our understanding of the link between superparasitism and population sex ratio. *N*. *vitripennis* is a model organism linking parasitism behaviour to individual brood sex ratio using the conceptual framework of local mate competition [50–53]. Where females of this species oviposit singly they produce *c*.80% daughters, in accordance with Local Mate Competition theory (LMC). In contrast, a superparasitising female will lay a male-biased brood to exploit the fitness opportunity from males being rare in the local mating pool. This effect is well established at an individual level in *N*. *vitripennis* under laboratory and field conditions [50,51] and is expected to generate a relationship between high superparasitism levels and more equal population sex ratio in natural populations [37,54]. Conversely, we have demonstrated that superparasitism promotes the spread of male-killing *A*. *nasoniae* through the population, which will act to inhibit the return of the population sex ratio towards parity. By facilitating the transmission of a sex-ratio distorting parasite, superparasitism reduces and destabilizes the male frequency compared to uninfected lineages, which would adhere to LMC predictions.

We also observed that high rates of superparasitism created a new condition under which male-killers could drive host extinction. Theoretical work has implicated sex ratio distortion in host extinction [52], including male-killing and feminizing endosymbionts [55,56], but has suggested they would only cause extinction where VT was near perfect and males mate globally (rather than locally). This possibility has been verified in laboratory populations of *Drosophila innubila*, where male-killing *Wolbachia* under strong VT conditions and local sibling competition caused host extinction due to virginity [41]. In our experiment, we observed population extinction when fly host resources are scarce and the male killer is driven to high prevalence through wasp superparasitism. Thus, the requirement of perfect VT for population extinction is relaxed when superparasitism occurs because mixed-mode transmission allow rapid spread of the microbe before deleterious virginity effects manifest at the population level.

Superparasitism can impact key ecological and evolutionary traits of a species. We show for the first time that this oviposition behaviour can drive remarkable changes in symbiont epidemiology, with consequences for host sex ratios and even host population survival. Additionally, these findings illuminate a previously unconsidered facet of the evolution and ecological impact of parasitoid superparasitism behaviour. Furthermore, our results challenge a major assumption in existing models; that heritable symbiont dynamics and the evolution of reproductive manipulation phenotypes are largely shaped within a framework of VT. In light of these data, we should work to incorporate the occurrence and frequency of mixed-mode transmission into a framework of symbiont epidemiology and host-symbiont interactions to capture the dynamics of symbionts such as *A*. *nasoniae*.

## Methods

### Experimental system

Arsenophonus nasonia is a γ-proteobacteria originally found infecting the parasitoid wasp Nasonia vitripennis where it distorts the secondary sex ratio towards a female bias by killing c.80% of male embryos [23,27,30]. VT efficiency of A. nasoniae in N. vitripennis has been estimated at 95% [23] and so generates a relatively minor rate of segregational loss which has been supposed, but not demonstrated, to be offset through fitness compensation through male-killing. Surveys of A. nasoniae in natural populations of N. vitripennis have found that among population infection prevalence varies between 5–47% [31,32], and several populations have failed to show any infection [31,32]. The factors causing this variation are unknown, but it can be assumed that there are barriers preventing its spread to certain populations to the high prevalences observed in some other male-killer systems [e.g. 57].

*N*. *vitripennis* is a model organism for sex ratio research and has been shown to adhere to LMC theory predictions in both laboratory and natural studies [36,50,53]. *N*. *vitripennis’s* utility as a study organism for sex ratio research has stemmed from its haplodiploid sex determination system. Female wasps can produce haploid sons from unfertilized eggs and so can alter their clutch sex ratio by controlling sperm access to their ova. Superparasitism is common in natural populations of *N*. *vitripennis* with up to 40% of broods founded by multiple mothers and up to nine mothers can contribute offspring to a single pupa [37,38]. The wasp’s dipteran hosts are typically aggregated around bird’s nests and animal corpses and so encourage high densities of wasps to congregate [38]. HT of *A*. *nasoniae* is readily achievable due to the per-oral transmission of the bacterium from maternal calyx fluid to offspring gut, therefore all larvae present in the host can acquire the infection [23,30]. Previous work has demonstrated that this HT is possible through interspecific multiparasitism, with success negatively correlated with genetic distance between species pairs [24].

The *A*. *nasoniae* strains used in *N*. *vitripennis*—only density experiments derive from wild caught *N*. *vitripennis* from Canada isolated by Graeme Taylor in 2010 [32], (CAN1). The male killing efficiency of this strain in *N*. *vitripennis* is *c*74% (see S1 Fig). The *A*. *nasoniae* used in multispecies experiments was isolated from an infected *N*. *vitripennis* caught in Marbury, Cheshire, UK by the authors (UK1). The male-killing efficiency of UK1 is given in S2 Fig and S2 Fig. Both strains were cultured on GC media supplemented with 3ml/L IsoVitalex (Applied Biosytems) at 25°C. All wasp lines were derived from isofemale laboratory cultures originally isolated in the Netherlands by the group of Leo Beukeboom. Wasps had been maintained, *A*. *nasoniae*-free for at least one year prior to experimentation. All wasps were maintained on *Sarcophaga bullata* pupae at 25°C, 12:12 L:D. *S*. *bullata* were obtained as larvae, allowed to pupate at 20°C and then either presented to wasps within 48 hours of pupation, or kept as pupae for up to one week at 4°C before use.

### Experimental procedure

*A*. *nasoniae* infected lines of *N*. *vitripennis* were established by injecting 5μl of *Arsenophonus* cells suspended in PBS at 10^5^ CFU ml^-1^ into a surface-sterilized fly pupa and then allowing female wasps to oviposit. Offspring from these broods were then allowed to mate and oviposit individually before being screened for infection as below. The F2 offspring of infected females were then established as infected (A+) isofemale lines kept *en masse*. A+ lines had been maintained in this way for at least two generations before experimentation. The uninfected line (A-) was retained as a comparator.

Infection prevalence in experimental populations was scored by diagnostic PCR following the protocol of [24]. Briefly, DNA was extracted from whole wasps using Chelex 100 [58]. PCR amplification specific to *A*. *nasoniae* based on the 16S ribosomal RNA gene was used to detect infected individuals (primers: **Arse16S**–F:GGG TTG TAA AGT ACT TTC AGT CGT/**Arse16S-R**: CGC AGG CTC GCC TCT CTC [1]). Approximately 1% of DNA samples in the first set of *N*. *vitripennis*-only experiments, showed inconclusive positive amplification of the Arse16S amplicon (weak or under/over sized bands). To correct for false positives these were further verified by performing an additional screen for the more specific, but type-II error prone, metallaprotease-1 gene (primers M1-F: GGGTCACATACCTATTTT, M1- 473 R: GTAGTCGCCTGGGTGGG, (GenBank accession: CBA72251.1, [52]). In all cases this verified that Arse16S primers had correctly identified an infected individuals and so this verification step was not used when screening samples from the multiple-species experiments. DNA quality was verified for each sample by amplifying a portion of the insect cytochrome oxidase gene (CO1), (Primers: **LCO**. 5' GGT CAA CAA ATC ATA AAG ATA TTG G 3, **HCO**. 5' TAA ACT TCA GGG TGA CCA AAA AAT CA 3' [59]), or the insect 18S rRNA gene (**NSF4/18:** CTG GTT GAT YCT GCC AGT, **NSR399/19**: TCTCAGGCTCCYTCTCCGG) [60]. Any samples that failed to amplify a CO1 product visible through gel electrophoresis were discarded from further analysis. When necessary, DNA samples were stored for short periods at -20°C or, for periods >1 week, at -80°C

### 1. Does manipulating superparasitism opportunity affect *A*. *nasoniae* dynamics in *N*. *vitripennis*?

#### Is superparasitism required for symbiont maintenance in N. vitripennis?

To test the hypothesis that superparasitism is required for *A*. *nasoniae* spread and maintenance in populations of *N*. *vitripennis* we established replicated populations of 80 female wasps under either enforced solitary parasitism conditions or permitted superparasitism.

Each population was subdivided into ‘patches’ consisting of isolated glass vials (75mm length × 25mm diameter) containing fly pupae for oviposition and capped with cotton wool. Solitary and superparasitism treatments had either one or four female wasps added to them respectively. Patches were then subdivided within parasitism treatments to either low (single fly pupa) or high (four fly pupae) resource categories, resulting in a 2×2 factorial design. The choice of superparasitism intensity and resource availability used here represent the natural extreme found by [37], wherein <10% of wild fly pupae four foundresses contribute offspring. Laboratory data comparing rates of production of Hamilton vs Fisher sex ratios from individual pupae estimates that *c*73% of pupae exposed to four wasps are subject to superparasitism under our experimental regime (see supplementary materials).

The parental generation of wasps used to establish populations were 50:50, *A+*:*A-*, with infected females distributed evenly across all patches within a population. Wasps were allowed to develop to adulthood and mate within their vial for 2–3 days post-eclosion. All wasps were then pooled and population sex ratio was estimated by sexing 100–150 individuals at random. Eighty females were then haphazardly allocated to fresh vials to propagate the next generation. A further 20 females were screened for infection. If the population size reduced below the 100 females required for this then the individuals used to propagate the next generation were reclaimed after 3 days of ovipositing and then screened. This design was intended to mimic the within-brood mating dynamics of a parasitoid with flightless males and dispersing females.

Control populations were run in parallel under the same demographic conditions but with no *A*. *nasoniae* infection in order to directly compare sex ratio. All populations were replicated six times and propagated for a maximum of eight generations. Replicate treatment populations were discontinued if two consecutive generations with 0% infection were observed–confirmed by screening 40 females in the second 0% generation When analysing population survival, these are considered extant on the basis that no uninfected control population went extinct. Populations that produced no wasps or single-sex broods were allowed to go extinct. The experiment was run in two blocks overlapping by one week, each containing three replicates of each population. All replicate control populations were maintained until all of their contemporary (within experimental block) infected populations were removed or the experiment ceased.

#### Does symbiont prevalence correlate with population-level superparasitism frequency?

In natural populations the intensity of superparasitism pressure is likely to be heterogeneous across patches within wasp populations [37,38]. We test the hypothesis that *A*. *nasoniae* prevalence will correlate with the level of superparasitism within a population. As before, replicated populations starting at 50% *A*. *nasoniae* infection were established, but the percentage of patches where superparasitism was permitted was manipulated to either 0%, 10%, 20%, 30%, 50%, and 100%. Populations consisted of 40 female wasps and infected individuals in the parent generation were evenly distributed across patches. Populations were propagated for four generations as described in the first experiment, with the exception that the offspring from all patches were allowed panmictic mating for 24 hours before exposure to new hosts. Following mass mating, 20 females were screened for infection prevalence. Each experimental population was run in triplicate.

### 2. Is *A*. *nasoniae* persistence associated with superparasitism in multiple parasitoid species?

Here, we test whether relatedness of a wasp species to *N*. *vitripennis* predicts *A*. *nasoniae* dynamics and determine the biological basis of the observed pattern.

#### Does phylogeny predict symbiont persistence?

We set up six replicate populations of four additional wasp species; *Nasonia giraulti*, *Nasonia longicornis*, *Trichomalopsis sarcophagae* and *Muscidifurax raptorellus*, and two lines of *N*. *vitripennis*, infected with the same clone of *A*. *nasoniae*. We established patches within the populations similar to the first experiments; here we had eight patches of five females with five pupae provided to each patch, per population. Patches were pooled in cell culture flasks prior to mass mating. In the first generation only single populations could be created for *N*. *longicornis*, *T*. *sarcophagae* and *M*. *raptorellus*. These were split in the second generation to create the final six populations. Following four generations we screened between 27–125 females (see supplementary Data [62]) from each population to determine the prevalence of *A*. *nasoniae*.

#### Does vertical transmission efficiency and cost of infection alone capture *A*. *nasoniae* dynamics in multiple species?

Within a VT-only framework interspecific variation in symbiont persistence can be due to two core factors: 1) differences in VT efficiency; 2) differences in cost/benefit of the male-killer infection. We measured how both VT rates and direct fitness effects of symbiont infection varied between the species. For each species we isolated 50 females from every population of the second generation of the experiment described above. These females were allowed access to two filth fly pupae for three days with no opportunity for superparasitism. After three days the fly pupae were split into separate vials, and the maternal wasps were removed and subject to PCR to ascertain *A*. *nasoniae* infection status.

To estimate VT efficiency, up to five offspring from a single fly pupa per maternal wasp were removed ~2 days post emergence (2 pupae = 10 offspring per wasp) and screened for infection to determine VT efficiency. To estimate the cost/benefit of symbiont infection the number of daughters produced by mothers that screened positively for *A*. *nasoniae* was compared to uninfected controls. This was not possible for *T*. *sarcophagae* due to a population crash in the uninfected controls. Broods were excluded from both VT and cost analyses if they contained less than two female offspring or if the mother was negative for *Arsenophonus* infection.

We used our observed levels of relative daughter production and VT efficiency to parameterize a minimal epidemiological model of *A*. *nasoniae* dynamics under VT-only. For this purpose we model only infection prevalence in female wasps because infected males do not transmit the bacterium and are thus epidemiologically inconsequential. First, we let the production of infected female offspring be given by:

$$P_{t,i}v_{i}(1+f_{i})$$

Where *P* is the *A*. *nasoniae* infection prevalence of females in generation *t* for species *i*, *v* is the VT efficiency of *A*. *nasoniae* in species *i* and *f* is the number of daughters produced by infected females expressed as a differential proportion of the number produced by uninfected females. This allows for infection to either increase or decrease female fecundity depending upon the sign of *f*: positive *f* would be classical fitness compensation whilst negative *f* denotes a cost of symbiont carriage.

We then express the number of infected females produced as a proportion of the total number of female offspring produced by both infected and uninfected females at time *t* to give the infection prevalence at *t* + 1.

$$P_{t+1,i}=\frac{P_{t,i}v_{i}(1+f_{i})}{\left(1-P_{t,i}+(P_{t,i}(1+f_{i})\right)}$$

We iterated this model for each species for four generations and then compared the model predictions with our observed *A*. *nasoniae* G4 prevalence in populations that were allowed to superparasitise. If A. nasoniae persists in these species through VT alone, then our model predictions should match our observed prevalence levels.

#### Do species vary in their superparasitism behaviour?

If HT is important for *A*. *nasoniae* dynamics then a species’ natural propensity to superparasitise may correlate with observable symbiont prevalence when the opportunities for superparasitism are available. Here, we test for differences in the propensity to superparasitise between the five parasitoid species. Females of each species were isolated under CO_2_ anesthetization, transferred to vials, given a host fly pupa and allowed to recover for several hours in order for CO_2_-induced paralysis to wear off. Half of the females were given new host pupae and observed for oviposition behaviour for 30 minutes before being removed. 3–4 hours subsequently the pre-parasitised pupae were transferred to vials holding single females from the second half of the cohort and again oviposition behaviour was observed for 30 minutes. Superparasitism was scored positively if a pupa was oviposited into by both females and avoidance scored if only the first female parasitized the host. Filth fly parasitoids typically share a similar pattern of behaviours when assessing host suitability before oviposition. We scored host acceptance when the distinctive abdominal arching during envenomation was observed as this trait has been shown to almost always precede oviposition [61]. Due to differences in generation time, not all wasp species could be observed simultaneously. As a standardized control, several *N*. *vitripennis* females were used in each session to determine whether there were any differences between sessions. Sample sizes for these behavioural assays are given in S1 Table.

### Statistical analyses

All statistical analyses were conducted in R using the lme4, fBasic, binom and multcomp packages (R Core team 2014). Where measured, sex ratio was recorded as the proportion of males in a clutch/population. Analyses of proportional data such as sex ratio and prevalence of *Arsenophonus* were carried out using generalised linear models with assumption for binomial distribution of errors and a logit link function. Where necessary, experimental block was included as a random factor (experiment set 1). Any overdispersion was accounted for by fitting either quasibinomial in GLMs or an observation-level random effect to account for high residual variation in GLMMs. All models were simplified to the minimum adequate form through pairwise maximum likelihood tests and AIC selection. Unless otherwise stated, statistics reported for fixed effects are generated by comparing models with/without the given effect. Multiple comparisons of factor levels within significant main effects were conducted with Tukey HSD obtained from the *‘multcomp’* package. Where applicable, Fisher’s exact tests and exact binomial tests were used to compare observed responses to predicted values or probability distributions. All data used in analyses and figures are available in the Dryad repository (doi:10.5061/dryad.60ff8) [62].

## Supporting Information

S1 Table Interspecific differences in superparasitism rates. Shown are the p-values obtained using pairwise comparisons. Significant contrasts in superparasitism rates shown in bold. Numbers in brackets = (Number of superparasitism events / number of trials).
(XLSX)

S2 Table Estimation of superparasitism rates by *N*. *vitripennis* under single and grouped conditions. Pupae exposed to four female wasps produced significantly fewer clutches with a female-biased sex ratio than pupa exposed to a single female wasp (comparison of binomial GLMER, χ^2^ = 17.601, df = 1, P<0.001). In total, 73% of pupae exposed to multiple wasps appear to be superparasitised. Clutches were classed as female biased if they showed a significant deviation from a 1:1 sex ratio in favour of males in exact binomial tests (Null hypothesis set to 0.5, significance cut off 0.95). Any clutch that showed no significant deviation from 1:1 was classed as 'not biased'. Unusually small clutches (<6 wasps) and those producing all male broods (indicative of maternal virginity) were removed from analysis.
(XLSX)

S1 Fig Male-killing efficiency of *A*. *nasoniae* strain (CAN1) used in all population experiments involving *N*. *vitripennis* only. Frequency distribution of sex ratio in clutches produced by 58 uninfected and 48 *A*. *nasoniae* infected *N*. *vitripennis*. *A*. *nasoniae* infection resulted in a significant decrease in male development (74% male death, comparison of binomial GLM χ^2^ = 109.06, df = 1, *P*<0.001). Wasps were derived from the same A+ and A- stocks used in the main study. Infection status of putatively A+ mothers was confirmed with PCR screening following oviposition.
(EPS)

S2 Fig Male-killing efficiency of the *A*. *nasoniae* strain (UK1) used in the multi-species population experiment. Frequency distribution of clutch sex ratio produced by isolated female *N*. *girualti*, *N*. *longicornis*, *N*. *vitripennis* and *M*. *raptorellus* originating from infected (light bars) and uninfected (dark bars) populations at generation 2 of the experiment. *A*. *nasonaie* infection was associated with a significant reduction in male offspring in all species (*N*. *girualti*: 28.3%, χ^2^ = 18.88df = 1, P<0.001, *N*. *longicornis*: 54.2%, χ^2^ = 12.64, df = 1, *P*<0.001, *N*. *vitripennis*: 60.9%, χ^2^ = 8.53 df = 1, *P* = 0.003, *M*. *raptorellus*: 94.2%, χ^2^ = 9.62 df = 1, *P* = 0.002). Infection status of putatively A+ mothers was confirmed with PCR screening post-oviposition, clutches with fewer than 6 wasps were excluded from analyses.
(TIFF)
