# Supplementary figures and images for: Superparasitism Drives Heritable Symbiont Epidemiology and Host Sex Ratio in a Wasp
Source: PLoS Pathog. 2016 Jun 20;12(6):e1005629. doi: 10.1371/journal.ppat.1005629 (PMC4920596; doi:10.1371/journal.ppat.1005629)

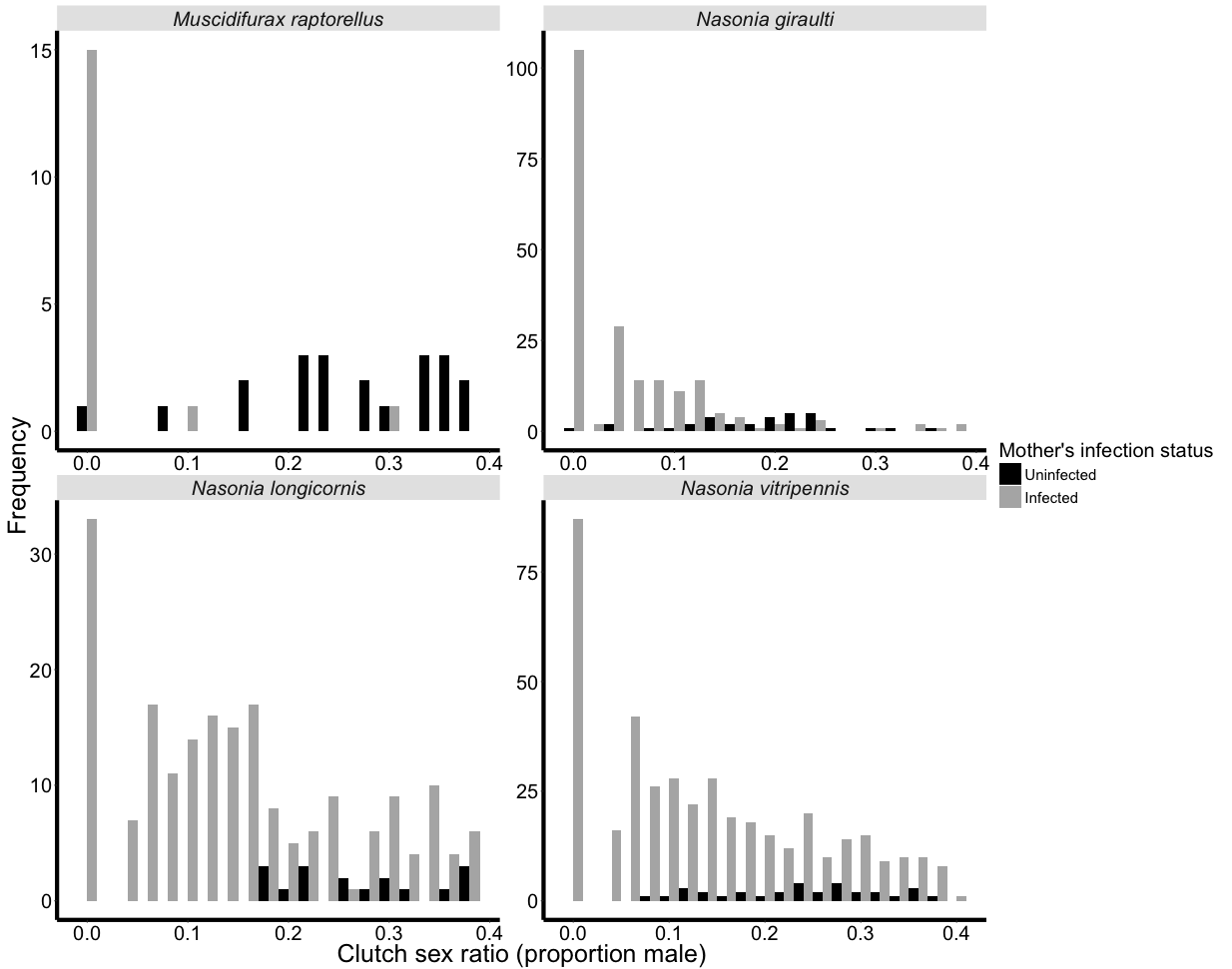

Supplement: S2 Fig — Frequency distribution of clutch sex ratio produced by isolated female N. girualti, N. longicornis, N. vitripennis and M. raptorellus originating from infected (light bars) and uninfected (dark bars) populations at generation 2 of the experiment. A. nasonaie infection was associated with a significant reduction in male offspring in all species (N. girualti: 28.3%, χ2 = 18.88df = 1, P<0.001, N. longicornis: 54.2%, χ2 = 12.64, df = 1, P<0.001, N. vitripennis: 60.9%, χ2 = 8.53 df = 1, P = 0.003, M. raptorellus: 94.2%, χ2 = 9.62 df = 1, P = 0.002). Infection status of putatively A+ mothers was confirmed with PCR screening post-oviposition, clutches with fewer than 6 wasps were excluded from analyses. (TIFF) [file ppat.1005629.s004.tiff]
